# MLVA typing of *Haemophilus influenzae* isolated from two Iranian university hospitals

**Published:** 2018-02

**Authors:** Faranak Nejati, Abolfazl Fateh, Seyed Ali Nojoumi, Mohammad Rahbar, Ava Behrouzi, Farzam Vaziri, Seyed Davar Siadat

**Affiliations:** 1 Department of Mycobacteriology & Pulmonary Research, Pasteur Institute of Iran, Tehran, Iran; 2 Microbiology Research Center (MRC), Pasteur Institute of Iran, Tehran, Iran; 3 Department of Microbiology, Reference Health Laboratories Research Center, Ministry of Health and Medical Education, Tehran, Iran

**Keywords:** *Haemophilus influenzae*, VNTR, MLVA

## Abstract

**Background and Objectives:**

Different serotypes of *Haemophilus influenzae* is now divided into 2 divisions: encapsulated and unencapsulated. Multiple locus variable number tandem repeat analysis (MLVA) includes such specifications as the extra power of separation, ease of data interpretation, and epidemiological data accordance, which have made it an appropriate molecular device for good typing and phylogenetic analysis of bacterial pathogens.

**Materials and Methods:**

In this research, cultured samples were studied and strains identified through biochemical tests were recognized. Moreover, DNA was extracted and studied qualitatively and quantitatively. Four pairs of specialized primers related to *H. influenzae* variable number tandem repeats (VNTR) and preparation of PCR were designed according to the regulated program. Also, electrophoresis of PCR products was performed. Finally, the interpretation of electrophoresis gel was done with respect to the observable bands showing the presence or absence of the required sequence in the samples related to every primer.

**Results:**

This study was the first MLVA typing of the unencapsulated *H. influenzae* in Iran. In this research, the VNTR sequences were tested in 30 strains without the unencapsulated *H. influenzae*. Among 30 mentioned strains, for which MLVA profile was obtained in this research, 25 different MLVA types were observed. Likewise, there was no repetition in VNTR sequences resulting from PCR in few *H. influenzae*. In all these cases, the number of repetitions in MLVA profile was determined as 0, except for one of the primers in 4 strains, which was 16%. However, this did not occur for the other VNTRs.

**Conclusion:**

The highest diversity of the repeats was for VNTR5 (7 types), followed by VNTR6 with 6 types of repeats, and VNTR12-1 and VNTR12-2 with 3 different types.

## INTRODUCTION

*Haemophilus influenzae* is a fastidious Gram-negative coccobacillus (1) and is classified into 2 typeable and nontypeable (NTHi) groups based on the presence or absence of capsules. The bacterium based on a capsule polysaccharide is divided into 6 sero-types (a, b, c, d, e, f), among which the clinical significance of serotype b is more than the rest (1–3). The genetic diversity of nontypeable strains is more than encapsulated strains (4). Encapsulated strains mainly cause invasive, systemic, and disseminating infections, such as bacteremia, pneumonia and acute bacterial meningitis caused by *H. influenzae* type b (Hib), while strains without capsule (NTHi) usually cause such infections as sinusitis in children, otitis media, respiratory tract infections, chronic bronchitis, or cystic fibrosis (5–9).

Since symptoms are often nonspecific, especially in pediatric patients, diagnosis of bacterial meningitis in most cases is seemingly difficult. Nowadays, to identify bacteria, new molecular techniques with both higher sensitivity and specificity with no need for the organism being alive are developed. PCR is among these techniques. Generally, a comparison between culture and PCR indicates that the PCR technique has a 99% sensitivity and specificity of culture. Due to the necessity of early detection and treatment of meningitis, utilization of this method is highly recommended.

In 2005, typing MLVA (Multiple Loci VNTR Analysis) for *H. influenzae* was invented by Schouls et al. (10, 11). MLVA is based on a small subset of variable VNTRs used to provide better resolution more than the most current methods of molecular typing (12–15). MLVA is an ideal subtyping tool for usual analysis of the number of isolates in the study of diseases (16). Multilocus VNTR analysis has the advantages of extreme resolving power, robustness, high throughput data portability, ease of data interpretation, and concordance with epidemiological data (17, 18). Another advantage is that unlike pulse-field gel electrophoresis (PFGE), MLVA is a PCR-based approach in which only a small amount of DNA is needed for analysis. Thus, the isolation of a live bacteria to measure MLVA is not obligatory. Polymorphic loci are applied to a number of variable TR loci (VNTR), which may have 2 to 10 alleles within a population. Therefore, combined multiple VNTRs can theoretically create a large number of genotypes (19). A wide range of mutations among VNTRs produces a wide range of allelic variation, and these markers are useful to evaluate the genetic relationship among different strains during periods of evolutionary time (20, 21). Based on this, MLVA can be a specific tool for molecular typing and phylogenetic analysis of bacterial pathogens (16, 22).

MLVA has been designed to provide better analysis of PFGE and distinguish similar samples of bacterial pathogens. MLVA has extreme resolving power for close isolates; it is also widely used for short-term epidemiological purposes including studies focusing on the outbreak of diseases and tracking the source of the pathogen and disease surveillance (23–26).

Perhaps, MLVA would not be abandoned in the near future, unless the whole genome sequencing and the final typing methods are both affordable and increasingly abundant. Because MLVA is a cost-effective and high-throughput subtyping tool, one can expect that further numbers of MLVA protocols for completing PFGE protocols be standardized for disease surveillance (16). In this study, we used MLVA typing for discrimination of *H. influenzae* isolates of Iran for the first time.

## MATERIALS AND METHODS

### Sample collection.

In this study, 30 strains of *H. influenzae* were collected from the eye, throat, respiratory tract, and cerebrospinal fluid of patients, who referred to Imam Khomeini and Milad hospitals in Tehran during 2012 and 2014.

### Phenotypic and genotypic confirmations.

Biochemical tests was used before DNA extraction to ensure the presence and growth of *H. influenzae*. The best way is the 24- hour culture (27) and different tests including oxidase and catalase (positive), urea, indole, and ornithine decarboxylase (positive or negative). The morphology of samples was checked with Gram staining method. The genotypes confirmation was performed as described previously (28). To confirm species identification, PCR amplification was performed to detect the *omp6* gene from genomic DNA (29).

Cell mass was maintained at −20°C for future use to prevent contamination; Cryo tubes with some of the samples were frozen at −70°C as well in case new and repeated subculture were to be used.

### DNA extraction.

DNA samples were extracted using Sinaclon DNA Extraction Kit (CinnaGen, Tehran, Iran), then, the isolated DNA was examined using a spectrophotometer (Nanodrop, Thermo Fisher Scientific, Carlsbad, CA, USA).

### PCR protocol.

To identify the strains VNTR sequences, primers of the Schouls’ study were taken and analyzed. The 25 μL reaction mixture contained 0.5 μL of each reverse and forward primer, 1 μL of DNA template, 12.5 μL of Master Mix (MgCl_2_, dNTPs, buffer and Taq polymerase), and the remaining volume contained double distilled water (DDW) (10).

**Fig. 1.**
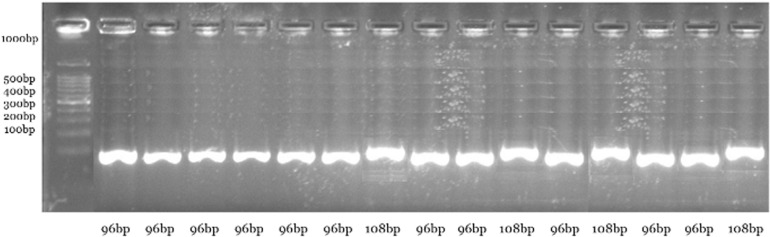
PCR amplification, Lane 1: Molecular weight marker, Lane 2-16: Gene PCR product

PCR process involves initial denaturing at 95°C for 15 minutes, then, 30 thermal cycles including 3 denaturing steps at 95°C for 30 seconds, whereby primers bind to the template strand at 50°C for 30 seconds and elongation amplified fragments at 72°C for 1 minute. The final amplification step was performed at 72°C for 10 minutes (10). The PCR products were separated electrophoresis on 2% agarose gel and amplified fragments were observed by UV illumination (Fig. 1). Based on the observed bands, the presence or absence of the target sequences in every one of the samples was interpreted compared to the marker. After obtaining the results of the electrophoresis related to all samples, the size of the produced band was measured by every sample in comparison to the marker.

### Statistical analysis.

Discriminating index (DI) as a numerical index is used to calculate the discriminative power of MLVA types. The following formula is used to calculate DI:

$$D=1-\frac{1}{N(N-1)}\sum_{J=1}^{s}x_{i(x_{i}-1)}$$

In this formula, N is the total strains used in MLVA typing, S stands for total number of different patterns, and X_j_ is the number of strains belonging to the type j.

## RESULTS

In this study, we examined VNTR sequences in 30 encapsulated *H. influenzae* strains. Among the 30 strains of *H. influenza* from which we obtained MLVA profiles in the present study, 25 different MLVA templates (83%) were observed compared to just 12% difference, which was observed in Schouls et al.’s study. The changes of VNTR loci in 30 strains of *H. influenzae* are presented in Table 1. In several strains of *H. influenzae*, results of PCR VNTR sequences revealed that they contain no duplicates. In such case, the number of repetitions in profile MLVA was considered as 0, which occurred in case of primer VNTR12-1 in 4 strains (16%), but it did not occur in other VNTRs. The highest diversity of repeats belonged to VNTR 5 (7 types), VNTR6 (6 types of repeats), followed by VNTR12-1 and VNTR12-2 with 3 different types. Based on what was reported in the Schouls’ study for VNTR5, repeat diversity was 9, VNTR6 had 7 repeat diversity, VNTR12-1 had 5 repeat diversity, and repeated variations observed in the VNTR12-2 was 9.

**Table 1.** Changes of VNTR loci in 30 strains of *H. influenzae*

**Number of repeats**	**Primers**			

	**VNTR5**	**VNTR6**	**VNTR12-1**	**VNTR12-2**
0			4	
7				2
8			12	20
9			14	8
14		2		
15		8		
16	3	1		
17	1	9		
18	5	8		
20	10	2		
21	3			
22	7			
23	1			

## DISCUSSION

Various repeat regions were identified in the full genome sequence of *H. influenzae* (30, 31). The length of the VNTRs was a stable genetic marker for separate colonies derived from a single clinical specimen or strains passages in the microbiology laboratory (32). To date, numerous VNTR sequences for *H. influenzae* have been mentioned in researches. Based on previous studies, we found the published sequences in Scholus’ study to be the most reliable. In this study, 30 samples were investigated using 4 VNTRs published in Scholus’ study.

When several strains isolated from different patients during an outbreak caused by *H. influenza* were analyzed, limited variation was encountered in VNTR site analysis (31). The highest number of repetitions (23 times) related to the VNTR5 was found in one of the strains; however, in the Schouls’ study, the number of repetitions was 16 times in 1 strain. Similarly, in VNTR5 in 7 strains and in each one 22 occurrences were observed. In the case of VNTR5, in most of the strains (10 strains), 20 repetitions were observed in each of them, and the remaining repeats were 16 (in 3 strains), 17 (in 1 strain), 18 (in 5 strains) and 21 (in 3 strains). However, compared to Schouls’ study, the number of repetitions was 2. For the analysis of *H. influenzae* included in the present study, the discrimination index (DI) of MLVA for VNTR5 was 0.81.

In VNTR6, the greatest number of repetitions was 20 reps, which occurred in 2 strains, in the next ranks, there were 8 in 18 strains and 17 in 9 strains. Others observed in VNTR6 were 14 reps (in 2 strains), 15 r (in 8 strains), and 16 reps (in 1 strain) compared to Schouls’ study, in which the highest number of repetitions in the VNTR6 was 2 (DI = 0.78).

In VNTR 12-1, the largest number that occurred in the 14 strains was 9, and 8 reps occurred in 12 strains, while when compared to Schouls’ study, most repeats in this VNTR were 4. In case of VNTR12-1 in 4 strains, there was no band in the electrophoresis results of PCR, and the number of repetitions was reported 0 (DI = 0.62).

The greatest number of repetitions on VNTR 12-2 was 8 in 20 strains, followed by 9 repeats in 8 strains and 7 repeats in 2 strains, while in Schouls’ study, the most repeat was 1 (DI = 0.49).

According to the discrimination index analysis, VNTR5 is the best one.

In contrast to Van Belkum research (33) in which they noticed multiple bands in the gel for some strains, this did not occur in the present study. There was an equal number of repeats in all the strains about VNTRs sizing 3bp, 5bp, and 6bp in their study. The highest number of repeats that occurred in 4bp VNTR was 40, but in the present study, the highest one was 23 repeats, which occurred in only 1 strain for VNTR5. The lowest number of repeats in their study occurred in 5bp VNTR in all the strains with 2 repeats; however, in the present study, 7 repeats were observed in VNTR12.

In another study by Van Belkum (31), repeats more than 60bp were observed for their VNTR4-10. However, this high repetition was not observed in our study. In one of their 4bp VNTRs, they noticed multiple bands in 2 strains (20%) as well. There were also 2 and 3 bands with a high frequency in their results. The lowest number of repeats in their study was 2 observed in one of 4bp VNTRs. In VNTR4-6, only 6bp repeats were observed in their study, inducting low variety in that VNTR.

In N. Randers study (32) like ours, no bands were observed in the cases. In their results for VNTR4-11, they found several high repetitions of 50bp, while 23bp band was observed only once in our study. For 3bp VNTRs, most repeats, for which high variability of VNTR is not shown, was 9; this occurred in our study for 12bp VNTRs with more variety. Unlike previous studies mentioned earlier, they surprisingly, even noticed one repeat of VNTR sequences, while they saw 2 bands in gel for one case.

In 30 strains of nontypeable *H. influenzae*, 25 different MLVA types (Table 2) were detected. The dominant type of MLVA was in Type 11 (13.3%), 8 (6.6%), 12 (6.6%), and 13 (6.6%).

**Table 2.** Composition and frequency of MLVA profiles in 30 strains of *H. influenzae*

**MLVA type**	**Frequency among strains**		**Number of repeats in VNTRs**		

	**All (NTHi)**	**VNTR5**	**VNTR6**	**VNTR12-1**	**VNTR12-2**
1	1	18	15	8	8
2	1	22	18	9	7
3	1	20	15	9	9
4	1	16	20	9	8
5	1	18	18	8	8
6	1	18	20	8	8
7	1	17	17	9	8
8	2	22	17	9	8
9	1	20	15	9	8
10	1	23	18	8	7
11	4	20	17	8	8
12	2	21	18	9	9
13	2	22	15	9	9
14	1	22	14	9	8
15	1	18	15	0	8
16	1	18	17	0	8
17	1	16	16	0	8
18	1	16	15	8	8
19	1	20	18	9	9
20	1	22	14	8	8
21	1	21	15	0	8
22	1	20	18	8	8
23	1	20	17	8	9
24	1	22	17	9	8
25	1	20	17	8	9

Comparing the MLVA type and its isolation site of the human body, it was found that among the strains with similar MLVA, Type 11 of MLVAs was separated from respiratory tract in 75% of the cases based on Table 2. Also, among different types of MLVA with less repeat frequency, including MLVA Type 12 in the table, they were all taken from the throat. MLVA Type 13 was also taken from the respiratory tract, while in MLVA Type 8, no special relationship was noticed. With respect to the low number of samples based on the above date and type of MLVA could not be established and further extensive studies should be conducted in this regard.

In conclusion, we could classify the strains based on anatomical site. We had 30 strains of NTHi that showed 25 patterns. However, the correlation of the subject and the isolation site require a larger sample size.
